# Two-Dimensional ABS_4_ (A and B = Zr, Hf, and Ti) as Promising Anode for Li and Na-Ion Batteries

**DOI:** 10.3390/molecules29215208

**Published:** 2024-11-04

**Authors:** Shehzad Ahmed, Imran Muhammad, Awais Ghani, Iltaf Muhammad, Naeem Ullah, Nadeem Raza, Yong Wang, Xiaoqing Tian, Honglei Wu, Danish Khan

**Affiliations:** 1 College of Physics and Optoelectronic Engineering, Shenzhen University, Shenzhen 518060, China; iltaf.muhammad@szu.edu.cn (I.M.); naeeman259@szu.edu.cn (N.U.); 2 Department of Chemistry and Guangdong Provincial, Southern University of Science and Technology, Shenzhen 518055, China; imrankhan@sustech.edu.cn; 3 Smart Materials for Architecture Research Lab, Innovation Center of Yangtze River Delta, Zhejiang University, Hangzhou 314100, China; awaisghani@zju.edu.cn; 4 Chemistry Department, Imam Mohammad Ibn Saud Islamic University (IMSIU), Riyadh 11623, Saudi Arabia; nadeemr890@gmail.com; 5 School of Physics, Nankai University, Tianjin 300071, China; yongwang@nankai.edu.cn; 6 National Laboratory of Solid-State Microstructures, Nanjing University, Nanjing 210093, China; 7 College of New Materials and New Energies, Shenzhen Technology University, Shenzhen 518118, China

**Keywords:** DFT study, ternary sulfides, two-dimensional materials, electronic structure, Li and Na ion storage, binding energy, ion diffusion barriers

## Abstract

Metal ion intercalation into van der Waals gaps of layered materials is vital for large-scale electrochemical energy storage. Transition-metal sulfides, ABS_4_ (where A and B represent Zr, Hf, and Ti as monolayers as anodes), are examined as lithium and sodium ion storage. Our study reveals that these monolayers offer exceptional performance for ion storage. The low diffusion barriers enable efficient lithium bonding and rapid separation while all ABS_4_ phases remain semiconducting before lithiation and transition to metallic states, ensuring excellent electrical conductivity. Notably, the monolayers demonstrate impressive ion capacities: 1639, 1202, and 1119 mAh/g for Li-ions, and 1093, 801, and 671 mAh/g for Na-ions in ZrTiS_4_, HfTiS_4_, and HfZrS_4_, respectively. Average voltages are 1.16 V, 0.9 V, and 0.94 V for Li-ions and 1.17 V, 1.02 V, and 0.94 V for Na-ions across these materials. Additionally, low migration energy barriers of 0.231 eV, 0.233 eV, and 0.238 eV for Li and 0.135 eV, 0.136 eV, and 0.147 eV for Na make ABS_4_ monolayers highly attractive for battery applications. These findings underscore the potential of monolayer ABS_4_ as a superior electrode material, combining high adsorption energy, low diffusion barriers, low voltage, high specific capacity, and outstanding electrical conductivity.

## 1. Introduction

Acquiring high energy and power densities in electrochemical storage devices is essential for developing portable electronics. The reliability of electrode materials is crucial for the performance of lithium-ion batteries (LIBs), which have become the dominant technology in energy storage [1]. Innovative materials are essential to satisfy the growing need for high energy and power density. Although graphene is a key electrode material, its theoretical capacity of 372 mAh/g restricts its capability to fulfill the increasing power density requirements [2,3,4,5]. Electrochemical energy storage systems are highly favored technologies today, with applications ranging from smart devices and electric vehicles to extensive power grids [6,7,8]. Consequently, advancing the renewable energy economy hinges on developing efficient, cost-effective, eco-friendly electrochemical energy storage solutions [9,10,11]. Particularly, the van der Waals (vdW) gaps in layered materials are crucial for the advancement of energy storage [12,13,14,15]. These voids facilitate effective ion intercalation and diffusion, increase conductivity and capacity, and promote stable phase transitions, all of which are critical for the advancement of high-performance, long-lasting next-generation batteries [16,17,18,19]. Delve into the groundbreaking domain of multilayer nanostructures, such as transition-metal dichalcogenides (TMDs) [20,21]. Two-dimensional (2D) materials, such as molybdenum disulfide (MoS_2_) and tungsten disulfide (WS_2_), provide notable benefits, including a large surface area, short diffusion routes, cost efficiency, and outstanding electrical conductivity [22,23]. Doping and alloying TMDs, such as synthesizing Mo_1−x_W_x_S_2_ alloys, enable accurate adjustment of their crystal structures and electrical characteristics, providing a viable method for improving performance in many applications [24,25]. Advanced battery technology requires electrochemically better ternary metal sulfides to enhance performance and efficiency. NiCo_2_S_4_ and CuFeS_2_ compounds are appropriate for sodium-ion and lithium-ion batteries due to their high conductivity and attractive redox characteristics [26,27,28]. In contrast, MnNi_2_S_4_ and CoMn_2_S_4_ enhance energy density and cycling stability, making them suitable for hybrid systems and energy storage [29,30,31]. Recent tailored nanostructures, especially nanoparticle and porous designs, can improve these sulfides’ electrochemical performance. Studies on MnNi_2_S_4_ emphasize sodium-ion intercalation, advancing battery material’s effectiveness and durability [32]. Ternary transition metal sulfides (TTMSs) are enabling next-generation energy storage solutions that are efficient and sustainable [33,34].

Recently, Gennevieve Macam and colleagues identified a range of TTMSs, specifically ABS_4_ (with A and B being Zr, Hf, and Ti) [35]. TTMSs can be synthesized using numerous processes, each offering unique advantages. Single Source Precursors (SSPs) provide exact regulation of phase and composition [36], while hydrothermal and solvothermal techniques are environmentally sustainable and economically efficient [37]. Mechanochemical synthesis facilitates scalability, while chemical vapor deposition (CVD) is optimal for producing high-quality films [38]. Colloidal synthesis guarantees uniform dispersion, while solid-state flux or salt melt techniques yield well-crystallized compounds [39]. 

In the present work, we employ first-principles calculations to explore the theoretical functionality of ABS_4_ materials as anode materials in alkali metal ion batteries. Initially, we confirmed the stability of the pristine ABS_4_ (with A and B being Zr, Hf, and Ti) monolayer by assessing its thermodynamic stabilities. We subsequently evaluated the adsorption behavior of Li/Na-ions on the ABS_4_ surface by analyzing their adsorption energies, charge density differences, and Bader charges. Furthermore, the climbing-image nudged-elastic-band (CI-NEB) approach was employed to assess the diffusion energy barriers associated with various Li/Na-ions pathways on the ABS_4_ monolayer. Finally, the voltage analysis and theoretical specific storage capacity were computed for Li/Na-ions on monolayer ABS_4_.

## 2. Computational Methods

We conducted all computations using the Vienna ab initio Simulation Package (VASP) based on Density Functional Theory (DFT) and Projector Augmented Wave (PAW) methods [40,41]. The electron–ion interaction and electron–electron exchange-correlation are investigated using the Perdew–Burke–Ernzerhof (PBE) function while maintaining a fixed cutoff energy of 550 eV for the plane-wave basis [40]. Geometric optimization is achieved using a conjugate gradient algorithm for force and energy, with thresholds set at 10^−3^ eV/Å and 10^−6^ eV. Approximately ± 25 Å of a vacuum slab was introduced along the z-axis to prevent interactions between periodic images. We employ non-spin-polarized calculations, a method widely used in similar research [42,43,44,45]. The van der Waals (vdW) interaction is incorporated for ion adsorption in the structure using the semiempirical Grimme D3 method [46]. We employed a Gaussian smearing method with a broadening parameter set to a specific value (e.g., SIGMA = 0.1 eV) to ensure proper integration over the electronic states. The Monkhorst–Pack scheme is also utilized with a mesh size of (14 × 8 × 1) for unicell and (5 × 3 × 1) for 2 × 2 × 1 supercell [47]. The diffusion pathways and energy barriers for Li/Na ions on the ABS_4_ monolayer were calculated using the climbing image nudged elastic-band (CI-NEB) method [48]. Additionally, the adsorption behavior of Li and Na on the ABS_4_ substrate is examined using the same mesh size (5 × 3 × 1). At the same time, the Bader method is employed to analyze charge transfer from the metal atoms to the ABS_4_ atoms. To assess thermal stability, ab initio molecular dynamics (AIMD) simulations are performed within an NVT utilizing the Nose–Hoover thermostat for temperature control, maintaining a stable 300 K and 500 K for 10 ps, employing a time step of 1 fs within a 6 × 4 × 1 supercell [49]. This method provides a reliable thermal environment for investigating the dynamic behavior of ABS_4_.

## 3. Results and Discussion

**Figure 1:**
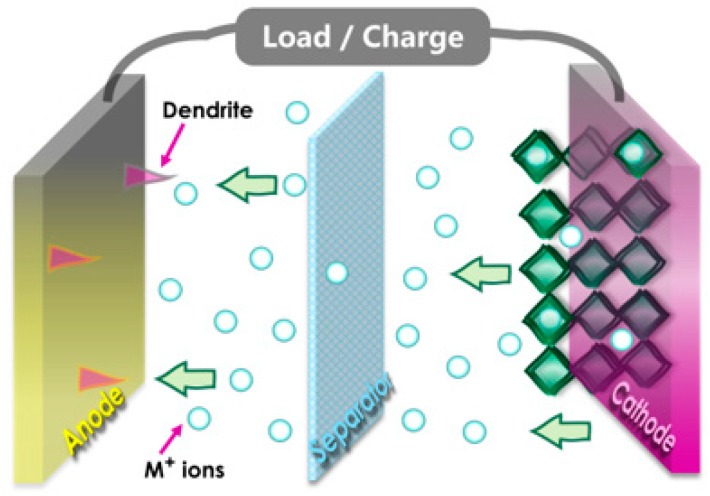
The Schematic illustrates the key elements, including the anode, cathode, and separator, and the flow of ions and electrons during charge and discharge cycles.

**Figure 2:**
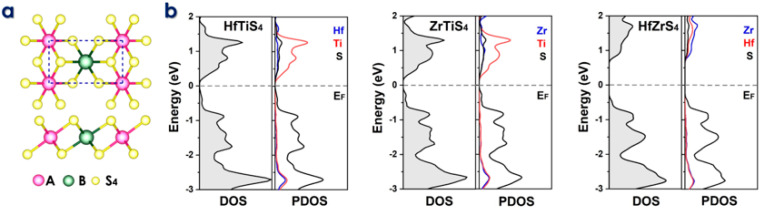
(**a**) Top and side views of the two-dimensional ABS_4_ structure, where A and B represent Zr, Hf, and Ti. (**b**) Density of States (DOS) and Partial Density of States (PDOS) plots for HfTiS_4_, ZrTiS_4_, and HfZrS_4_, illustrating their electronic properties.

In the schematic design of conventional batteries, the intercalation and deintercalation of ions between electrodes are mechanisms by which they operate, as shown in Figure 1. The movement of electrons and ions is involved in the charge and discharging processes. The 1T phase has preference over the 2H phase for ABS_4_ due to its superior electrochemical properties, which include higher ion capacities, enhanced conductivity, and lower diffusion barriers [50,51,52]. Consequently, it is the optimal choice for high-performance anode applications [53]. Furthermore, 1T-ABS_4_ enhances hydrogen evolution catalytic performance, accelerates charge/discharge cycles, facilitates efficient electron transport, and provides superior thermal stability for high-temperature and energy storage applications [54]. Similarly, as illustrated in Figure 2a, monolayer ternary transition metal sulfides (TTMSs) can form three combinations in a monoclinic structure with the chemical formula ABS_4_, where A/B = Zr, Hf, or Ti. TTMSs are derived from group IVB transition metals (Ti, Zr, Hf) and chalcogen elements (S). The 1T-phase AS_2_ unit cell was enlarged into a 2 × 1 supercell, and one transition metal atom was swapped out for an element to construct the atomic structure [55]. Using this method, the structure of ABS_4_ can be adjusted to match the configuration of ABS_4_, producing three distinct compounds, e.g., HfTiS_4_, ZrTiS_4_, and HfZrS_4_. TTMSs are surrounded by six sulfur atoms, resulting in a triangular prismatic or octahedral coordination geometry. TMSs are classified into multiple phases, with 1T and 2H being the most prominent, based on the layering arrangement of ABS_4_ layers and this coordination. In the present work, we considered 1T-phases; the measured lattice constants of 1T-phases are a = 6.100, 6.141, 6.314 Å, and b = 3.523, 3.544, 3.644 Å, for HfTiS4, ZrTiS4, and HfZrS_4_ consistent with findings from previous studies [54].

The electronic characteristics of TTMSs can be better understood using Density of states (DOS) and partial density of states (PDOS) analysis. They reveal the distribution of electronic states at the Fermi level, which is essential for predicting conductivity and electrochemical performance. DOS reveals energy gaps and conducting behavior, while PDOS shows how certain atomic orbitals (e.g., metal or sulfur atoms) affect electronic states, improving understanding of ion adsorption and charge transfer. We evaluated DOS, which refers to a narrow bandgap HfTiS_4_ (0.18 eV), ZrTiS_4_ (0.21 eV), and HfZrS_4_ (1.12 eV). Partial density of states (PDOS) analysis highlights the total and varying contributions of different elements (Hf, Ti, Zr, and S) to the electronic states near the Fermi level in the ABS_4_. These contributions show how each element affects TTMS’s electronic structure, as shown in Figure 2b. Additionally, the thermal stability of the ABS_4_ monolayers was evaluated using finite temperature molecular dynamics calculations at temperatures including 300 K and 500 K, as depicted in Figure S1. The ab initio molecular dynamics simulations confirm the high stability of these structures. Performance analysis of ABS_4_ as a potential anode material for Li/Na- ion batteries. The primary criterion for battery application is the adsorption strength of metal ions on a substrate. Therefore, our initial investigation uses the following equation to determine this adsorption strength.

*E**_ads_*** = (*E**_Mx(ABS_******_4)_*** − *E**_ABS_******_4_*** − *xE**_M_***)/*x* (1)

**Figure 3:**
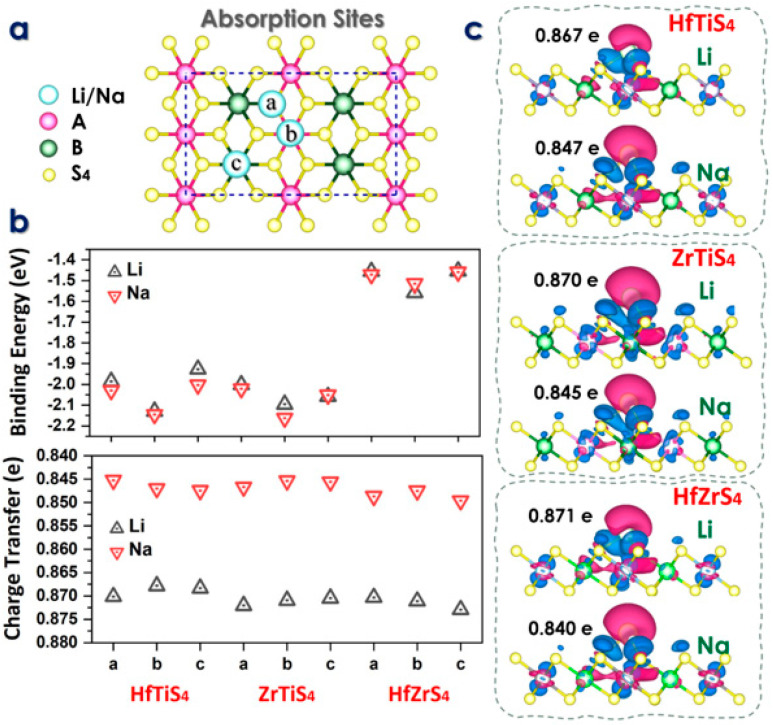
(**a**) Schematic illustration of three absorption sites. (**b**) Binding energy at each absorption site and charge transfer analysis (**c**) Charge density difference maps at the stable adsorption sites for Li and Na ions in HfTiS_4_, ZrTiS_4_, and HfZrS_4_.

In the above equation, *E**_Mx(ABS_******_4)_*** is the total energy of the ABS_4_ monolayer with adsorbed Li/Na, *E**_ABS_******_4_*** is the intrinsic total energy of the 2 × 2 × 1 ABS_4_ supercell, and *E**_M_*** is the energy per atom derived from the bulk material of Li/Na. According to the ABS_4_ geometric symmetry, three distinct adsorption sites, a, b, and c for Li/Na-ions, labeled as follows: above the A atom (b), above the B atom (c), and the S atom (a), as shown in Figure 3a. 

Indicates that Li atoms prefer to adsorb at the b-site, with an energy of 2.12, 2.09, 1.55 eV, and 2.14, 2.16, 1.51 eV for Na-ions. The computed adsorption energy *E**_ads_*** of Li and Na on each site are given in Figure 3b. The ABS_4_ monolayers have adsorption energies between these values (Figure 3b). The results indicate that the ABS_4_ monolayers exhibit favorable adsorption energies. The findings suggest that ABS_4_ monolayers effectively inhibit the formation of dendrites or metal clusters on the electrode surface. The charge density difference between the Li/Na ions and ABS_4_ monolayers is studied with an iso-surface value of 0.004 e per Bohr^3^, where the green and red colors show the charge gain and loss, respectively (Figure 3c). The charge transfer of Li/Na-ions is illustrated through the calculation of the charge density difference, defined as

Δρ = ρ***_M(_*_ABS__4_*_)_*** − ρ***_M_*** − ρ***_ABS__4_*** (2)

whereas ρ***_M(_*_ABS__4_*_)_*** is the charge density of the alkali metal ABS4 monolayer, ρ_M_ is the charge density of a Li/Na-ion, and ρ_ABS4_ is the charge density of the ABS_4_ system. The findings are depicted in Figure 3c, where blue and red colors indicate charge gain and loss, respectively. These graphs show that the ABS_4_ monolayers accumulate charge while the Li/Na ions lose charge, suggesting that the ionized Li/Na atoms transfer their charges to the monolayers. The electrical characteristics using Bader charge analysis to measure Li/Na-ions charge transfer to the ABS_4_ monolayer. As previously found, Li/Na-ions transfer 0.86/0.84 |e|, 0.87/0.84 |e|, 0.87/0.84 |e| to site-b for HfTiS_4_, ZrTiS_4_, and HfZrS_4_, as shown in Figure 3c. 

**Figure 4:**
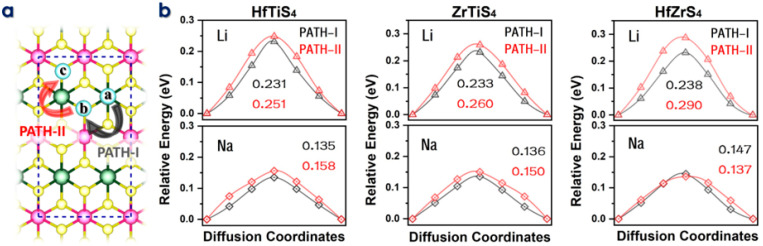
(**a**) Schematic illustration of two ion diffusion paths in HfTiS_4_, ZrTiS_4_, and HfZrS_4_. (**b**) Diffusion barriers for Li and Na ions in HfTiS_4_, ZrTiS_4_, and HfZrS_4_, including their corresponding energy barriers.

The rate of Li/Na-ions migration through the electrolyte and electrodes dramatically affects the performance of batteries. Materials with high electrical conductivity enable quick electron transfer and reduce diffusion lengths for lithium-ion migration, improving power capability. To migrate, intercalating ions must move. The climbing image nudged elastic band (CI-NEB) approach calculates diffusion barriers and minimal energy pathways (MEP) for Li/Na-ions migration on the HfTiS_4_, ZrTiS_4_, and HfZrS_4_ monolayers (Figure 4a). Figure 4b shows the migration path Li-ions, the energy barriers along PATH-I were 0.231 eV, 0.233 eV, and 0.238 eV for HfTiS_4_, ZrTiS_4_, and HfZrS_4,_ respectively. PATH-II showed barriers of 0.251 eV, 0.260 eV, and 0.290 eV. Similarly, for Na ions, the barriers for PATH-I were 0.135 eV, 0.136 eV, and 0.147 eV; and for PATH-II, they were 0.158 eV, 0.150 eV, and 0.137 eV for HfTiSe_4_, ZrTiS_4_, and HfZrS_4_, respectively. The diffusion barrier is low as compared to the largely reported anode material [55,56,57,58,59].

**Figure 5:**
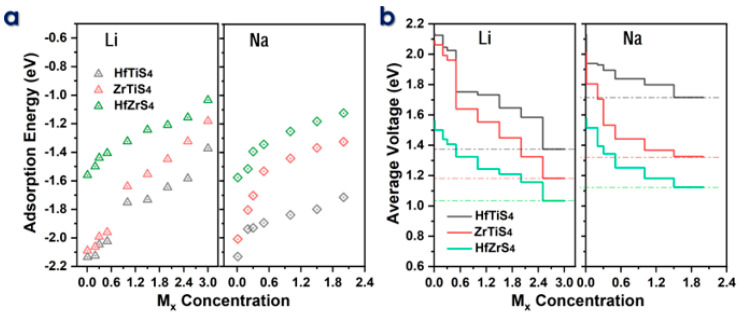
(**a**) Adsorption energy of HfTiS_4_, ZrTiS_4_, and HfZrS_4_ as a function of metal ion concentration. (**b**) Voltage profile for Li and Na ions as a function of metal ion content, illustrating their electrochemical behavior.

The electrochemical efficiency of batteries is the theoretical specific capacity (C) and average voltage (AV). The adsorption energies (E_ad_) of Li and Na ions on HfTiS_4_, ZrTiS_4_, and HfZrS_4_ were computed for ion concentrations ranging from 0.1 to 3.0 M_x_ for Li ions and from 0.1 to 2.0 M_x_ for Na ions (Figure 5a). We consider seven different Li/Na-ion concentrations for all structures to evaluate the maximum storage capacity; the binding energy decreases as the metal ion concentration M_x_ increases; the decrease in B.E is mainly due to the repulsive forces between neighboring ions. The theoretical capacity was calculated by using the following equation:

*C* = x*F*/3.6*M_ABS4_* (3)

In the equation, (x) represents the total number of adsorbed Li/Na ions in ABS_4_, while F denotes Faraday’s constant, which is 96,485.3329 s A mol^−1^, and (M) stands for the molar mass of ABS_4_, while 3.6 serves as a conversion factor related to the stoichiometry of ion intercalation. Specifically, the Li-ion capacities are 1639 mA/h, 1202 mA/h, and 1119 mA/h, and the Na-ion capacities are 1093 mA/h, 801 mA/h, and 671 mA/h for ZrTiS_4_, HfTiS_4_, and HfZrS_4_, respectively. The predicted capacity is much higher than that of well-known 2D anode materials [60,61,62,63]. Theoretically, capacity concerning concentration, as shown in Figure 5b, is utilized in the following equation to calculate the average voltage for a monolayer.

*V* = − [*E_MxABS4_* − *E_ABS4_* − x*E_Li/Na_*]/Z*xe* (4)

In the above equation, *E_ABS4_* and *E_MxABS4_* represent the energy of the ABS_4_ monolayer and the energy after Li/Na-ions adsorbed ABS_4_, respectively, *E_Li/Na_* is the energy of the Li/Na ions in the bulk system, and (x) indicates the total number of Li/Na ions adsorbed. Li-ions average voltages of 1.16 V, 0.9 V, and 0.94 V, whereas Na-ions have 1.17 V, 1.02 V, and 0.94 V for ZrTiS_4_, HfTiS_4_, and HfZrS_4_. All the average voltage values for Li and Na, as shown in Figure 5b, are within the acceptable voltage range. The calculated average voltages confirm that ABS_4_ is a suitable material for the anode, contributing to the high output voltage of the batteries [43,64,65].

**Figure 6:**
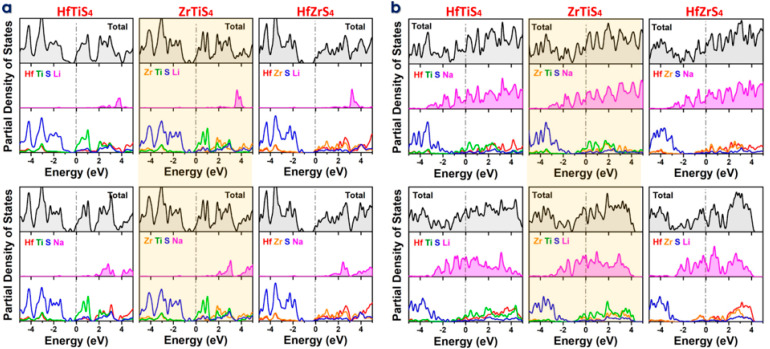
Density of States (DOS) and Partial Density of States (PDOS) for HfTiS_4_, ZrTiS_4_, and HfZrS_4_ (**a**) Absorption of a single Li/Na-ion. (**b**) Fully adsorbed Li/Na-ions.

The DOS and PDOS are crucial for understanding the electronic behavior of monolayers like HfTiS_4_, ZrTiS_4_, and HfZrS_4_ while adsorbing Li and Na ions. DOS and PDOS identify localized states near the Fermi level when a single ion is adsorbed, showing how it interacts with the host monolayer (Figure 6a). The PDOS shows hybridization between the ions orbitals (e.g., Li 2s/Na 2p) and those of the host atoms, helping explain electronic structure alterations, including band gap changes and conductivity shifts. DOS and PDOS indicate ion accumulation affects the ABS_4_ electronic structure in the completely adsorbed state, frequently causing larger energy changes and conducting behavior (Figure 6b). The results indicated that all the structures retain their metallic character for fully adsorbed structures. Understanding these ABS_4_ electrochemical activity, charge mobility, and ion storage capacity helps determine their viability for energy storage applications. Prospective areas of research were delineated, involving studies on alternative ion types and alterations to material characteristics for enhanced performance. Furthermore, it addresses problems associated with the experimental implementation of these materials, including scalability, stability, and the development of a solid-electrolyte interphase (SEI). Ternary metal sulfides exhibit substantial theoretical capacities and advantageous electrochemical properties; nonetheless, they encounter constraints that impede their application in battery technologies. It is essential to address stability, ion diffusion, structural integrity, and reversibility to improve the practicality and efficacy of these materials in contemporary battery systems. Additional research and development are essential to address these difficulties and enhance performance.

## 4. Conclusions

In conclusion, our study highlights the remarkable advancements in identifying high-performance anode materials for metal-ion batteries, focusing on ternary transition metal sulfides (TTMSs) analyzed through density functional theory (DFT). We investigated the ABS_4_ monolayer structures, where A and B are Hf, Ti, and Zr, revealing their exceptional potential as anode materials. Our first-principles analysis uncovered several critical advantages of these materials, including their metallic behavior and stability across a range of Li and Na adsorption concentrations. Notably, ABS_4_ monolayers exhibit impressive performance metrics: HfTiS_4_ stands out with the highest specific capacities of 1639 mAh/g for lithium and 1093 mAh/g for sodium. The average operating voltages are within the optimal range, at 1.16 V for lithium and 1.17 V for sodium. Furthermore, the low energy barriers of 0.231 eV for lithium and 0.251 eV for sodium facilitate efficient ion diffusion, leading to rapid charge and discharge capabilities. These findings underscore that ABS_4_ monolayers are promising candidates for high-performance anodes in lithium and sodium-ion batteries, combining high specific capacity, favorable voltage profiles, and swift ion transport. Our results advocate for the potential of ABS_4_ monolayers as superior materials for next-generation energy storage technologies.

## Data Availability

The data presented in this study are available upon request from the corresponding author.

## Supplementary Materials

The following supporting information can be downloaded at: https://www.mdpi.com/article/10.3390/molecules29215208/s1, Figure S1 Energy evolution and structural dynamics of ZrTiS4, HfTiS4, HfZrS4.
