# Supplementary material for: Two-Dimensional ABS4 (A and B = Zr, Hf, and Ti) as Promising Anode for Li and Na-Ion Batteries
Source: Molecules. 2024 Nov 4;29(21):5208. doi: 10.3390/molecules29215208 (PMC11547295; doi:10.3390/molecules29215208)
Supplement: Supplementary file 1 [file molecules-29-05208-s001.zip › molecules-3227519-supplementary.pdf]

Article

Two-Dimensional ABS_4 (A and B = Zr, Hf, and Ti) as Promising Anode for Li and Na-Ion Batteries

Shehzad Ahmed [1,*], Imran Muhammad [2], Awais Ghani [3], Iltaf Muhammad [1], Naeem Ullah [1], Nadeem Raza [4], Yong Wang [5], Xiaoqing Tian [1,6,*], Honglei Wu [1,*] and Danish Khan [7,*]

1 College of Physics and Optoelectronic Engineering, Shenzhen University, Shenzhen 518060, China; iltaf.muhammad@szu.edu.cn (I.M.); naeeman259@szu.edu.cn (N.U.)
2 Department of Chemistry and Guangdong Provincial, Southern University of Science and Technology, Shenzhen 518055, China; imrankhan@sustech.edu.cn
3 Smart Materials for Architecture Research Lab, Innovation Center of Yangtze River Delta, Zhejiang University, Hangzhou 314100, China; awaisghani@zju.edu.cn
4 Chemistry Department, Imam Mohammad Ibn Saud Islamic University (IMSIU), Riyadh 11623, Saudi Arabia; nadeemr890@gmail.com
5 School of Physics, Nankai University, Tianjin 300071, China; yongwang@nankai.edu.cn
6 National Laboratory of Solid-State Microstructures, Nanjing University, Nanjing 210093, China
7 College of New Materials and New Energies, Shenzhen Technology University, Shenzhen 518118, China
* Correspondences: ahmed@szu.edu.cn (S.A.); xqtian@szu.edu.cn (X.T.); hlwu@szu.edu.cn (H.W.); khandanish@sztu.edu.cn (D.K.)

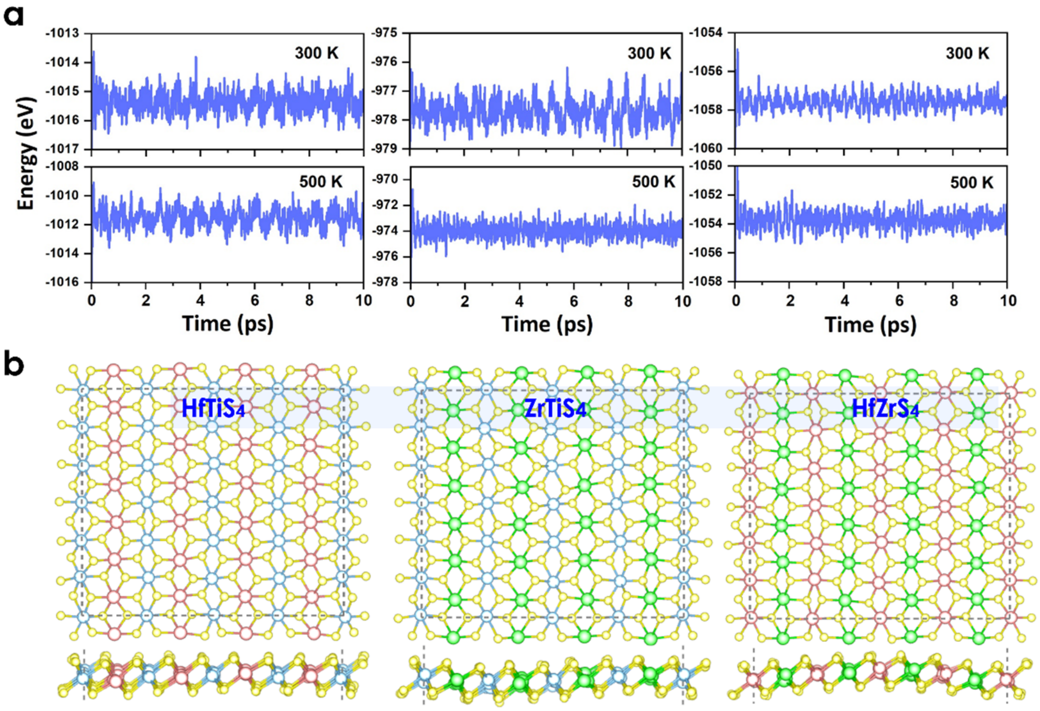

Figure S1. (a) The evolution of the system energy overtime during an ab initio molecular dynamics (AIMD) calculation at 300 K and 500 K. (b) A snapshot of the crystal structural dynamics at 500 K showcases the top view of $ZrTiS_4$, $HfTiS_4$, and $HfZrS_4$ crystal structures obtained during an AIMD simulation.
